# Ensuring sufficient service capacity for removals of long-acting reversible contraceptives: a mixed-method study of provider experiences in Senegal

**DOI:** 10.12688/gatesopenres.13600.1

**Published:** 2022-04-08

**Authors:** Aurélie Brunie, Megan M. Lydon, Salif Ndiaye, Fatou Ndiaté Rachel Sarr Aw, Elena Lebetkin, Alice Cartwright, Sarah Brittingham, Marème Dabo, Etienne Dioh, Marème Mady Dia Ndiaye

**Affiliations:** 1 FHI 360, Washington, D.C., USA; 2 FHI 360, Durham, NC, USA; 3 Centre de Recherche pour le Développement Humain (CRDH), Dakar, Senegal; 4 IntraHealth International, Dakar, Senegal; 5 Sénégal Ministère de la Santé et de l'Action Sociale, Direction de la Sante de la Mère et de l'Enfant, Division Planification Familiale, Dakar, Senegal

**Keywords:** Senegal, sub-Saharan Africa, family planning, contraception, contraceptive implant, intrauterine device (IUD), Long-acting reversible contraceptive (LARC), LARC removal

## Abstract

**Background: **As the number of implants and intrauterine devices (IUD) used in sub-Saharan Africa continues to grow, ensuring sufficient service capacity for removals is critical. This study describes public sector providers’ experiences with implant and IUD removals in two districts of Senegal.

**Methods: **We conducted a cross-sectional study with providers trained to insert implants and IUDs from all public facilities offering long-acting reversible contraceptives. Data collection elements included a survey with 55 providers and in-depth interviews (IDIs) with eight other providers. We performed descriptive analysis of survey responses and analyzed qualitative data thematically.

**Results: **Nearly all providers surveyed were trained in both implant and IUD insertion and removal; 42% had received training in the last two years. Over 90% of providers felt confident inserting and removing implants and removing IUDs; 15% were not confident removing non-palpable implants and 27% IUDs with non-visible strings. Challenges causing providers to refer clients or postpone removals include lack of consumables (38%) for implants, and short duration of use for implants (35%) and IUDs (20%). Many providers reported counseling clients presenting for removals to keep their method (58% implant, 31% IUD), primarily to attempt managing side effects. Among providers with removal experience, 78% had ever received a removal client with a deeply-placed implant and 33% with an IUD with non-visible strings. Qualitative findings noted that providers were willing to remove implants and IUDs before their expiration date but first attempted treatment or counseling to manage side effects. Providers reported lack of equipment and supplies as challenges, and mixed success with difficult removals.

**Conclusions: **Findings on provider capacity to perform insertions and regular removals are positive overall. Potential areas for improvement include availability of equipment and supplies, strengthening of counseling on side effects, and support for managing difficult removals.

## Introduction

Long-acting reversible contraceptives (LARCs), including implants and the copper intrauterine device (IUD), present positive characteristics, including high effectiveness, long duration of action, few contraindications, and reversibility with a prompt return to fertility ^1^. They do not depend on user adherence and do not require daily attention or pericoital use. Additionally, compared to short-acting methods like pills and injectables, LARCs require fewer visits for re-supply. At the same time, while developments with injectable contraceptives allow for self-injection in a growing number of countries ^2^, LARCs must be inserted by a trained provider. Implants and IUDs also require removal by a trained provider at the end of their labeled duration of use or at any time of the user’s choosing. As such, ensuring access to removals plays a critical role in informed choice in family planning ^3, 4^.

With the growing popularity of implants in sub-Saharan Africa, the need for removals is accelerating ^5^. Yet some data indicate that service capacity for implant removal may lag behind that for insertion ^5, 6^. While ease of removal has increased with 1- and 2-rod implant technologies compared to the six-rod Norplant systems, implant removals remain more technically demanding than insertions, especially in cases involving difficult removals such as when the implant was deeply inserted, has migrated, has broken, or has become encapsulated. Removal may also become challenging in cases where clients have gained or lost weight, making the implant more difficult to locate ^7^. Clinical challenges are closely related to programmatic challenges associated with scaling-up the availability of both implant insertion and removal services: providing sufficient training to health care providers, including in difficult removal techniques such that providers are confident in their removal skills; ensuring that appropriate equipment and supplies are available for both routine and difficult removals; and establishing necessary referral mechanisms as needed, including for difficult removals or where providers may insert but not remove implants ^5, 7^. A few recent studies which document the client perspective in relation to implant removals in sub-Saharan Africa reiterate these challenges ^8– 13^.

Compared to implants, IUD utilization tends to be lower in sub-Saharan Africa, even though copper IUDs have longer efficacy and have been available for decades. Recent targeted efforts to re-invigorate IUD use have often been focused on specific populations, including postpartum/postabortion users and integrated with HIV services, rather than the general population ^14^. This may begin to change as hormonal IUDs become more widely available, particularly in the public sector ^15, 16^. However, while service delivery patterns of IUD uptake have been documented in the literature, there are few studies describing women’s experiences seeking IUD removal ^8, 17^ or provider experiences providing care.

To our knowledge, few studies capture provider experiences providing implant and IUD insertions and removals in sub-Saharan African settings. Available evidence includes a study of medical and nursing students’ self-assessments of their ability to provide implants at the community level in the Democratic Republic of Congo ^9^ and qualitative studies documenting insufficient training, lack of equipment and time, and unclear referral pathways for difficult removals as barriers to providing implant removals in Botswana ^13^, lack of experience and lack of refresher trainings as barriers to inserting IUDs cited by midwives in Ghana ^18^, and insufficient space to provide IUDs mentioned by providers and stakeholders in Ethiopia ^17^. None of these studies offer a side-to-side comparison of provider experiences with implant and IUD removals.

This study conducted in Senegal assesses potential challenges with LARC removals from the perspective of providers to inform potential adjustments. Senegal was the first African country to introduce Norplant, starting with a pre-introductory trial in 1996 ^19^. Second-generation implants like Jadelle (2-rod levonorgestrel implant) and Implanon (now Nexplanon, a 1-rod etonogestrel implant), introduced in the country in 2009 and 2014, respectively, are currently available. As of 2017, implants represented 31% of the method mix and IUDs 8%, with over 90% of LARCs obtained through the public sector ^20^. The specific objectives of this study were to describe provider training and clinical experiences with implant and IUD removals.

## Methods

We conducted a cross-sectional, mixed-method study including a tablet-based survey and in-depth interviews (IDIs) with providers from all public sector facilities offering LARCs in two districts of Senegal (Dakar Centre and Kolda). The study was initially planned for three districts representing different geographic and cultural contexts; work in the third district was cancelled due to the onset of the coronavirus disease 2019 (COVID-19) pandemic. Providers who had been trained to insert implants and had been at their current facility for at least one year were eligible. We established a listing of all public facilities offering LARCs within the two districts and identified eligible providers within them. Sample size was driven by the number of facilities; we aimed to survey between one and two providers from all facilities offering LARCs depending on the number of providers that were eligible and available. Following evidence that 80% saturation can be achieved within 8 IDIs, we purposively selected four facilities per district that had at least two eligible providers and represented different levels of the health system (health centers and health posts) and invited one provider from each of these facilities to participate in an IDI, for a total of 8 providers ^21^.

The study team worked with facility managers to gain access to health facilities, identify all eligible providers, and introduce the study team to the providers. Senegalese female research assistants hired as consultants approached selected providers in-person and interviewed consenting providers individually and in private at health facilities in French between January 22 and March 18, 2020. Trained research assistants captured survey data on tablets; two separate research assistants with advanced degrees and prior qualitative experience conducted, audio-recorded, and transcribed IDIs in French. Research assistants recorded observations as field notes that were reported into the transcripts. Participants had limited knowledge about the research assistants and transcripts were not returned to participants for review.

Survey and IDI topics included training, client counseling at insertion and removal, and clinical experiences with insertions and removals. The survey also explored if providers had ever encountered specific obstacles to removals, while IDIs included additional themes related to equipment and supplies, referral dynamics, and fee structure. Both instruments included questions regarding difficult removals, defined as removal of non-palpable implants and of IUDs with non-visible strings. We developed the data collection instruments based on the existing literature and adjusted them through peer review and extensive discussions with Senegalese study staff and partners. To identify potential problems in the design of questions and likely response options, research assistants conducted cognitive interviews with 10 providers who met eligibility criteria but were recruited outside of the study area. The cognitive interviews were conducted using a rapid iterative approach, observed by one of the investigators (AB), and documented through notes. To inform final revisions prior to data collection, research assistants conducted a pre-test of both instruments with another set of providers with characteristics similar to those of study participants. The final study materials used can be found as *Extended data* ^22^.

Providers gave written consent prior to interviews. The Comité National d’Ethique pour la Recherche en Santé in Senegal (SEN19/65, approved November 7, 2019) and FHI 360’s Protection of Human Subjects Committee in the United States (1383816-2, approved June 19, 2019) approved the study.

We performed descriptive analysis of survey responses using Stata version 15 (RRID:SCR_012763). Typed transcripts were uploaded into NVivo 12 (RRID:SCR_014802) for coding and applied thematic analysis. Two analysts (MML and HD) coded transcripts using a codebook combining a priori codes identified based on informational needs and data-driven codes that emerged from the initial reading of transcripts, with periodic verification of intercoder agreement. Three analysts (MML, HD and VL) then developed analytic memos exploring patterns in the data.

## Results

### Quantitative results

We surveyed 55 providers from 35 facilities; an additional 7 facilities offered LARCs but had no eligible providers. All providers that were approached consented to participate. Providers were mostly women (76%) and midwives (62%), with an average of 11 years of experience in their current designation ( Table 1) ^22^.

**Table 1.** Provider characteristics and LARC training.

Characteristic, n (%)	n=55
**Female**	42 (76.4)
**Current designation**	
Midwife	34 (61.8)
Nurse	13 (23.6)
Nurse assistant	7 (12.7)
Health worker	1 (1.8)
**Years in current designation** (mean (SD))	10.6 (5.4)
**Training in implant provision**	
Insertion and removal	55 (100)
**Training in IUD provision**	
Insertion and removal	52 (94.6)
Insertion only	1 (1.8)
Neither insertion nor removal	2 (3.6)
**Currently offering implant insertions**	55 (100)
**Ever performed implant removal**	55 (100)
**Currently offering IUD insertions**	49 (89.1)
**Ever performed IUD removal**	46 (83.6)
**Timing of last training on implant** **removal**	
0–11 months	7 (12.7)
12–23 months	16 (29.1)
24–35 months	9 (16.4)
36+ months	20 (36.4)
Unsure	3 (5.5)
**Timing of last training on IUD removal**	
0–11 months	4 (7.3)
12–23 months	19 (34.6)
24–35 months	8 (14.5)
36+ months	19 (34.6)
Unsure	2 (3.6)
Not trained in removal	3 (5.4)

**Acronyms** **LARC – long-acting reversible contraception** **IUD - intrauterine device**

***Insertion and removal training and experience*.** All providers were trained in both implant insertion and removal and 95% were trained in IUD insertion and removal ( Table 1). All providers reported they offered implant insertions at the time of the survey and 89% IUD insertions. Additionally, all providers had ever removed an implant, and 84% had ever removed an IUD. Overall, 42% of providers had received training on implant removal in the last two years and 42% on IUD removal.

**Figure 1.**
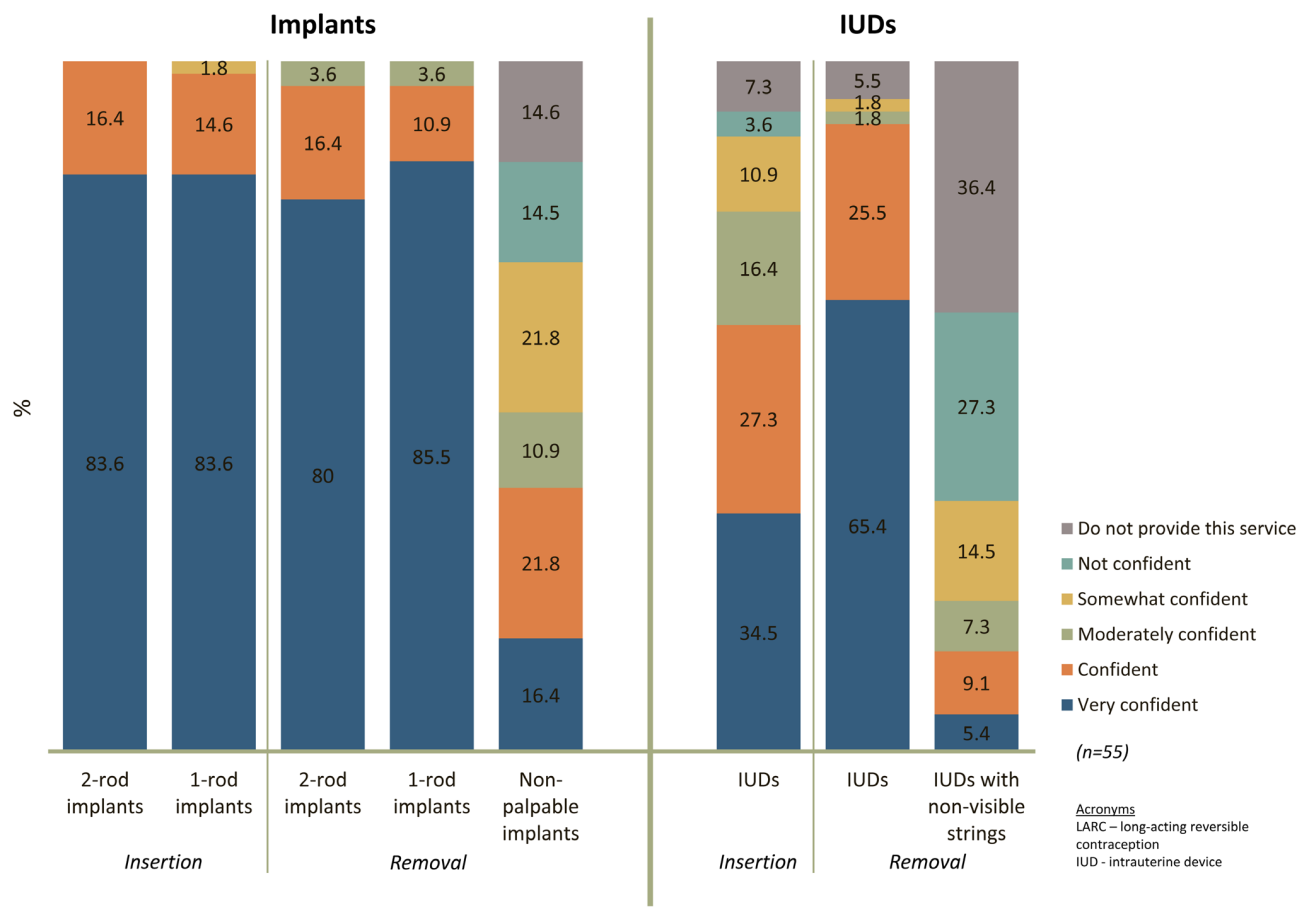
Self-reported confidence in inserting and removing LARCs, by method.

When asked to rate their confidence inserting and removing implants and IUDs, 96-100% of providers said they felt confident or very confident inserting and removing both Jadelle and Implanon ( Figure 1). However, 15% of providers indicated they did not feel confident removing non-palpable implants and an additional 15% said that they did not provide this service. For IUDs, 62% of providers felt confident or very confident when performing insertions and 91% when performing removals; 27% of providers said they were not confident removing IUDs with non-visible strings and an additional 36% said that they do not perform this service.

***Counseling at insertion*.** All providers reported that they were providing implant insertion services at the time of the survey, while 89% (n=49) reported they were providing IUD insertion services. Among these, 98% of implant providers and all IUD providers reported counseling clients on contraceptive-induced menstrual changes (CIMCs) and/or non-bleeding side effects at insertion ( Table 2). Most providers who counsel their implant clients on CIMCs and/or other side effects reported mentioning irregular bleeding (91%) and amenorrhea (82%) when inserting implants, with 65% mentioning weight gain and 56% headaches. For IUDs, most providers said they told their clients about irregular bleeding (57%), abdominal/pelvic pain (57%), and heavy bleeding (55%).

**Table 2.** LARC counseling provided at insertion among providers offering LARC insertions, by method.

n (%)	Among providers offering implant insertions (n=55)	Among providers offering IUD insertions (n=49)
**Counseling provided on contraceptive-induced** **menstrual changes (CIMCs)/side effects**		
CIMCs and non-bleeding side effects	53 (96.4)	44 (89.8)
CIMCs only	1 (1.8)	4 (8.2)
Non-bleeding side effects only	0 (0.0)	1 (2.0)
Neither CIMCs nor non-bleeding side effects	1 (1.8)	0 (0.0)
**CIMCs and non-bleeding side effects mentioned** ^a^	**n=54**	**n=49**
Irregular bleeding	49 (90.7)	28 (57.1)
Amenorrhea	44 (81.5)	7 (14.3)
Weight gain	35 (64.8)	-
Headaches	30 (55.6)	5 (10.2)
Heavy bleeding	24 (44.4)	27 (55.1)
Dizziness	21 (38.9)	1 (2.0)
Weight loss	19 (35.2)	-
Prolonged bleeding	13 (24.1)	12 (24.5)
Abdominal/pelvic pain	10 (18.5)	28 (57.1)
Nausea/vomiting	7 (13.0)	-
Infection	4 (7.4)	15 (30.6)
Menstrual cramps	-	16 (32.7)
**Gives at least one reason for removal**	41 (74.5)	35 (71.4)
**Reasons mentioned for removal** ^a^	**n=41**	**n=35**
Desired pregnancy	21 (51.2)	18 (51.4)
Non-bleeding side effects	15 (36.6)	14 (40.0)
CIMCs	11 (26.8)	7 (20.0)
Can remove for any reason	11 (26.8)	10 (28.6)
Implant/IUD expired	10 (24.4)	5 (14.3)
Weight gain	6 (14.6)	-
Increased/abnormal blood pressure	5 (12.2)	-
**Counseling provided on where to seek removals**		
This facility only	13 (23.6)	10 (20.4)
This facility and another facility	42 (76.4)	39 (79.6)

^a^ Among providers offering counseling. Multiple responses possible, with spontaneous responses; responses with values of ≥10% for one method reported. **Acronyms** **CIMCs - contraceptive-induced menstrual changes** **LARC – long-acting reversible contraception** **IUD - intrauterine device**

Altogether, 25% and 29% of providers providing implant and IUD insertion services, respectively, said they did not talk to their clients about reasons for removal at the time of insertion. Among those who did (n=41 for implants and n=35 for IUDs), the most common reason for which providers mentioned telling their client they could remove their method was desired pregnancy (51%), compared to 37–40% for non-bleeding side effects, 27–29% for “any reason,” 20–27% for CIMCs, and 14–24% for expiration of the method.

***Factors affecting provision of removals*.** When presented with a list of specific situations that they may have encountered during which clients requested LARC removal, 38% of all providers said they had ever referred or asked a client coming for an implant removal to come back due to lack of consumables, 35% due to the client only having the method for a short time, 33% due to the client arriving late to the facility at the end of consultations, and 26% due to being too busy ( Table 3). For IUD removals, the most commonly reported reason for not providing a removal was short duration of use (20%). In addition, 58% of providers said that they had ever counseled a client coming for an implant removal to keep their method, compared to 31% of providers for IUD clients requesting removal. Among providers who reported they had told a client it was better to keep her method (n=32 for providers with experiences with implants, and n=17 with experiences with IUDs), we asked their reasons for counseling clients to keep their method. The main reasons reported were attempting to manage side effects first, clients having had the method for a short time, and having clients weigh challenges experienced while using the method against a possible pregnancy ( Table 3). 

**Table 3.** Experiences referring or asking clients to come back for removals among all providers, by method.

n (%)	Among providers reporting experiences with implants (n=55)	Among providers reporting experiences with IUDs (n=55)
**Experiences referring clients or asking** **clients to come back for a removal** ^a^		
Told client better to keep method	32 (58.2)	17 (30.9)
Lack of supplies, like gloves, compresses, anesthesia	21 (38.2)	2 (3.6)
Client only had method for short duration	19 (34.5)	11 (20.0)
Client arrived late	18 (32.7)	3 (5.5)
Too busy to attend to client	14 (25.5)	2 (3.6)
Lack of equipment, like scalpel or forceps	9 (16.4)	2 (3.6)
Necessary equipment had not been sterilized	7 (12.7)	6 (10.9)
Did not feel confident performing the removal	5 (9.1)	3 (5.5)
Client unable to pay for services	4 (7.3)	0 (0.0)
**Reasons given to clients for keeping their** **method** ^b^	**n=32**	**n=17**
Should try to manage side effects first	29 (90.6)	14 (82.4)
Made client weigh keeping method against pregnancy risk	16 (50.0)	4 (23.5)
Had not had method long	14 (43.8)	8 (47.1)
Client did not want to get pregnant	3 (9.4)	1 (5.9)

^a^ Multiple responses possible; providers were probed about each situation ^b ^ Among providers who reported they had told a client it was better to keep her method. Multiple responses possible; spontaneous responses **Acronyms** **IUD - intrauterine device**

***Experiences with difficult removals*.** While all providers reported having removed an implant, 78% reported ever having a client present for a removal with a deeply placed implant, and 31% of those reported encountering this situation in the last three months ( Table 4). Altogether, 58% of providers said they had successfully removed a non-palpable implant at least once. Many providers had also successfully removed implants in clients with excessive weight gain (determined subjectively by provider) (75%), as well as implants with a broken rod (53%) or a bent rod (49%). Over a third had removed implants in situations with excessive bleeding during removal and with excessive rod encapsulation. Non-palpable implants were the most common scenario for referrals, reported by 16% of providers. 

**Table 4.** Experiences with difficult implant removals among providers who have ever removed an implant.

n (%)	n=55
**Ever encountered a situation where removing an implant was difficult** **because it was placed too deeply**	43 (78.2)
**Had a client present with a deeply placed implant in three months** **preceding the survey**	17 (30.9)
**Experiences successfully performing difficult removals** ^a^	
Client gained a lot of weight	41 (74.6)
Deeply inserted/non-palpable	32 (58.2)
Broken rod	29 (52.7)
Bent rod	27 (49.1)
Excessive bleeding during removal	19 (34.6)
Excessive rod encapsulation	19 (34.6)
Rod inserted somewhere else besides arm	3 (5.5)
**Experiences referring clients or telling clients to come back due to** **difficult removals** ^a^	
Deeply inserted/non-palpable	9 (16.4)
Excessive rod encapsulation	2 (3.6)
Client gained a lot of weight	1 (1.8)
Bent rod	1 (1.8)
Rod inserted somewhere else besides arm	1 (1.8)
Excessive bleeding during removal	0 (0.0)
Broken rod	0 (0.0)

^a^ Multiple responses possible; providers were probed about each situation

Among the 84% (n=46) of providers who had ever removed an IUD, 33% had experienced removal requests for IUDs with non-visible strings, and 22% had faced this situation in the last three months ( Table 5). Overall, 26% of those who had removed an IUD had successfully removed an IUD with non-visible strings and just 4% had ever successfully managed removal of an IUD embedded in the uterine wall. In comparison, 17% and 4% of providers who had ever performed IUD removal had referred clients elsewhere for these reasons, respectively.

**Table 5.** Experiences with difficult IUD removals among providers who have ever removed an IUD.

n (%)	n=46
**Ever encountered a situation where removing an IUD was difficult because** **of non-visible strings**	15 (32.6)
**Had a client present with an IUD with non-visible strings in three months** **preceding the survey**	10 (21.7)
**Experiences successfully performing difficult removals** ^a^	
Non-visible strings	12 (26.1)
IUD embedded in uterine wall	2 (4.4)
IUD perforated through uterine wall	0 (0.0)
**Experiences referring clients or telling clients to come back due to difficult** **removals** ^a^	
Non-visible strings	8 (17.4)
IUD embedded in uterine wall	2 (4.4)
IUD perforated through uterine wall	0 (0.0)

^a^ Multiple responses possible; providers were probed about each situation **Acronyms** **IUD - intrauterine device**

### Qualitative results

Of the eight IDI participants, seven were midwives. Respondents were equally divided across the two districts, and across health centers and health posts. IDIs lasted 75 minutes on average.

***Removal skills*.** Although nearly all respondents had received pre-service education covering implant and IUD removals, several mentioned that the training lacked opportunities for practice, with most participants first performing a removal during their internship. Several participants shared challenges relating to maintaining removal skills in service, including low client volume, especially of IUD users, and infrequent and irregular refresher trainings, particularly regarding difficult removals.

***Counseling at insertion*.** While several IDI participants noted that they tell clients they can remove their method at any time, a couple reported mentioning particular circumstances for which clients can return for removal before labeled duration of use including poor general health, desired pregnancy, and weight gain with implants, or increased bleeding with IUDs. A couple of providers also believed implants to have shorter efficacy among overweight clients. A nurse assistant from Kolda explained:

*For Jadelle, I often tell women weighing less than 80kg that they can keep their implant for 5 years. For those more than 80kg, now I tell them to come back after 4 years of use.* (49-2)

***Management of requests for removal before labeled duration of use*.** Several providers agreed that a desire for pregnancy or lack of sexual activity were particularly suitable reasons for removal before labeled duration. A nurse assistant from Kolda conveyed a sentiment shared by others, stating that, “ *when they get married after insertion, it’s really appropriate to remove it and have a child*” (49-2).

In response to requests for implant removal before labeled duration related to CIMCs or side effects, most providers reported first addressing misconceptions, providing reassurance counseling or attempting treatment, as well as engaging clients in considering the implications of discontinuing their method. Several described agreeing to removal before labeled duration only after first exploring other alternatives with the client. For example, one midwife in Dakar described their approach:

*I try to sensitize them about the risks of removal related to an unwanted pregnancy, now after discussion, if I find that the woman sticks with her decision, I become open to her request.* (69-1)

However, respondents widely agreed that implants should be removed for clients with high blood pressure or hypertension, explaining that it was a contraindication. While some providers recommended immediate removal for such clients, others described close monitoring with removal if high blood pressure persisted or became very elevated. A midwife in Kolda explained:

*There are also the eligibility criteria such as high blood pressure. If the blood pressure level is very high because of the implant, I remove it.* (53-1)

Similarly, most respondents reported advising removal before labeled duration to clients who were overweight or had gained weight. This included some perceiving implants had shorter efficacy among overweight women, and others explaining they were concerned about the negative health effects of obesity and therefore recommended removal to help clients manage their weight.

Regarding IUDs, several providers were hesitant to remove the method before labeled duration when clients complained about feeling the strings, either by themselves or by their partner during sex. For example, a midwife in Dakar Centre shared, “ *there are women who come and tell you, ‘it bothers my husband’, ‘my husband told me he can feel it,’ okay, in this case, we don't remove it*” (61-5). Instead, providers reported engaging in counseling, verifying the IUD is well placed, shortening long strings, and/or moving strings to the side. Participants had mixed approaches concerning sexually transmitted infections and other vaginal infections. Some felt the infections could be treated with the IUD in place while others described removing it prior to treatment and then replacing the IUD when the infection cleared or offering another method.

***Factors affecting provision of removals*.** All participants reported lack of supplies and equipment as a challenge in providing removals. Examples included lack of surgical pliers, scalpel blades, or ultrasound machines, as well as anesthetic and sterile gloves. Providers reported that clients were routinely required to purchase some consumables and expected to purchase additional products when there were stockouts.

Several respondents also described challenges around equipment sterilization. Some providers reported that frequent sterilization of limited numbers of tools increased wait times. Others described difficulties with dysfunctional sterilizers or intermittent power supply that sometimes resulted in referring clients or delaying removal.

***Experiences with difficult removals*.** Nearly all participants had encountered non-palpable or deeply placed implants. Participants reported mixed success attempting such removals, often describing the experience as “ *fishing*” for the implant rod. Providers explained that these cases took longer (from 30 minutes to upwards of an hour), caused clients more pain, could require re-application of anesthetic, and sometimes resulted in making a deep incision or multiple incisions in the client’s arm. Often, providers referred clients with non-palpable implants to places with imaging equipment to locate the rods. Participants expressed varied comfort levels with deep removals: one remarked they immediately refer such cases upon identification, others described attempting removal and then referring to a more experienced colleague or another facility, and some successfully removed difficult cases and even received referrals from colleagues.

Most participants had also encountered broken implants, with challenges including finding all the pieces and ensuring all had been removed. Providers described these cases as requiring greater “ *patience”* and skillful technique.

Most had confronted removal cases for IUDs with non-visible strings. Participants described the importance of access to ultrasound technology to manage these cases. Those with ultrasound access reported successful removals. Others attempted removal without it, with several providers reporting they subsequently referred clients to other facilities with ultrasound to complete the removal, such that a midwife in Dakar explained, “ *I realized that the strings weren’t there, I fished around in vain. Then too, I ended up referring to the district [hospital]*” (62-11).

## Discussion

We found that all providers who had been trained in implant insertion had also been trained on removal, and that all but one provider offering IUD insertion had received training on IUD removal. Moreover, close to half of providers had received some training on removals in the two years preceding the survey. Provider confidence with both implant insertion and removal was high, though qualitative interviews noted increased opportunities to practice removals during pre-service education and in service as potential areas for improvement. Confidence levels with implant insertion tended to be higher than for IUD insertion, and providers were overall more confident about IUD removals than IUD insertions. This finding may reflect inherent technical differences between procedures. In that sense, it is generally consistent with another study reporting on average time spent performing these different procedures in Nigeria, which found similar amounts of time required for inserting implants and IUDs (7–11 minutes), but more time to remove implants (14–21 minutes) compared to IUDs (4 minutes) ^23^. Additionally, this finding may be partly explained by the fact that demand for implants is higher than IUDs in the population overall, so providers have more regular opportunities to practice their skills. Innovations in the implant trocars, such as with Nexplanon, have made problematic deep insertions less likely ^7^. IUD insertions (and removals) may also require the need for more dedicated, private facility space, which was noted as a barrier to IUD provision in Ethiopia ^17^.

Current service guidelines in Senegal include guidance on side effect counseling at insertion but no recommendations that providers counsel on where and when to seek removals. While providers reported informing clients of where to go for LARC removal, our findings indicate that discussion on when to seek a removal, including the option to remove the method at any time, may be more limited. CIMCs and non-bleeding side effects are a key reason for discontinuation, including for LARCs ^24, 25^. While almost all providers offering LARCs reported counseling clients on CIMCs and other side effects, we found variability in the specific topics covered during counseling, leading to gaps in providing clients with comprehensive information. Counseling on irregular bleeding and amenorrhea, two likely CIMCs associated with implant use, was common but not universal; only a little over half of providers reported counseling IUD users on the likelihood of experiencing heavy bleeding, a common side effect with this method. High quality family planning counseling, including information on possible side effects and how to manage them, is associated with method continuation ^26, 27^. In addition to method benefits and mechanisms of action, counseling materials could be strengthened by the addition of comprehensive information on possible side effects and on the option to remove LARCs at any time. Including job aids such as the NORMAL tool should be considered when updating materials ^28, 29^.

Quantitative and qualitative findings indicate that many providers counseled clients seeking removals to keep their method. Qualitative findings further highlight differential attitudes toward various reasons for removals, with providers for example being inclined to remove implants when a client presents seeking pregnancy or with weight gain or hypertension but preferring to first offer reassurance counseling or treatment for CIMCs. Potential provider misconceptions about method eligibility warrant attention, such that providers discussed high blood pressure as a contraindication to implant use in qualitative interviews while this is not been substantiated by evidence ^30^. Reassurance by providers may contribute to acceptability of CIMCs ^31^; however, even if potential concerns around health effects can be alleviated, CIMCs can disrupt women’s domestic, religious, or work life, though some may see benefits to reduced bleeding, such as amenorrhea ^15, 31, 32^. Counseling job aids and other tools for reassurance counseling and side effects management when clients are interested in keeping the method should be made available. Messaging around voluntary discontinuation should also be reinforced through formative supervision and onsite coaching updates.

Our findings also provide some evidence of systemic challenges, including lack of consumables (particularly for implant removal) and providers being too busy to attend to clients. Similar constraints were documented through the experiences of implant users in Ghana ^10^. In addition, we found that providers may be reluctant to perform a removal for clients who only had their method for a short period of time. This perspective was also documented in other studies, potentially due to concerns about wastage of products ^10^. Systemic barriers and potential biases warrant attention as part of a comprehensive approach to support quality removal services and ensure full, free, and informed choice.

Currently limited evidence exists on difficult LARC removals in low- and middle-income country settings, though issues with nonpalpable/deeply inserted implants and broken or bent rods are not uncommon ^10, 33, 34^. In this study, about three quarters of providers had encountered removals involving deeply inserted implants and one third of providers who had ever removed an IUD had encountered removal cases with non-visible strings. Although many providers reported having successfully performed removals in these situations, there was also evidence of referrals. Quantitative findings indicate gaps in confidence compared to regular removals and qualitative interviews reveal some difficult experiences. More research is needed to understand the prevalence of difficult removals and quality and continuity of care for women seeking removals.

While this study brings valuable insights to improve removal services, some limitations must be acknowledged. A possibility of recall bias and courtesy bias exists whereby providers may report inaccurately based on their memory of past events or of what they may perceive as undesirable attitudes or behaviors. The selection of providers for this study is mainly purposive and not intended to support statistical generalizability; however, the results from this study provide evidence that can help program managers in Senegal identify challenges and opportunities for strengthening LARC service provision.

## Conclusion

This study conducted in two districts of Senegal illuminates many similarities in provider experiences with implant and IUD removal services and demonstrates strong capacity to manage regular LARC removals. The Direction de la Santé de la Mère et de l’Enfant at the Ministry of Health led the presentation of results to stakeholders in Senegal, including regional and district health teams and implementing partners. Several recommendations emerged from this dissemination, including strengthening counseling materials with anticipatory guidance on CIMCs and non-bleeding side effects, developing guidance on side effect management, emphasizing quality of care for removals through formative supervision and onsite coaching updates, and addressing gaps in the supply chain, notably around the availability of consumables. In order to ensure high quality care, support for management of difficult removals also warrants ongoing attention in Senegal and other countries.

## Data availability

### Underlying data

Full qualitative transcripts are not available for ethical reasons because even after removing directly identifiable information such as names, age, and facility, participant identity may be difficult to fully conceal, and research locations may remain potentially identifiable, presenting a risk of deductive disclosure. However, codebooks and relevant excerpts of transcripts are available from the authors on reasonable request. Requests should be sent to the corresponding author at elebetkin@fhi360.org. Requests will be granted to researchers for the purposes of comparative analysis, upon approval from relevant ethics committees.

Harvard dataverse: Access to Implant and IUD Removals in Senegal (AIIRS). https://doi.org/10.7910/DVN/DLW6F8 ^22^.

This project contains the following underlying data:

- Senegal_AIIRS_ClientInPerson.tab (data from client in person questionnaire)
- Senegal_AIIRS_ClientPhone.tab (data from client phone questionnaire)
- Senegal_AIIRS_Facility.tab (data from Health facility survey)
- Senegal_AIIRS_Provider.tab (data from provider questionnaire)

### Extended data

Harvard dataverse: Access to Implant and IUD Removals in Senegal (AIIRS). https://doi.org/10.7910/DVN/DLW6F8 ^22^.

This project contains the following extended data:

- Senegal AIIRS study_data documentation.pdf
- Senegal AIIRS_Facility Survey_codebook.pdf
- Senegal AIIRS_ICF_Clients_IDI.pdf
- Senegal AIIRS_ICF_Clients_survey in person.pdf
- Senegal AIIRS_ICF_Clients_survey phone.pdf
- Senegal AIIRS_ICF_Facility in charge.pdf
- Senegal AIIRS_ICF_Providers_IDI.pdf
- Senegal AIIRS_ICF_Providers_survey.pdf
- Senegal AIIRS_IDI guide_provider.pdf
- Senegal AIIRS_IDI guide_women.pdf
- Senegal AIIRS_Inperson survey women_codebook.pdf
- Senegal AIIRS_Phone survey women_codebook.pdf
- Senegal AIIRS_Provider Survey_codebook.pdf

Data are available under the terms of the Creative Commons Zero "No rights reserved" data waiver (CC0 1.0 Public domain dedication).
