# Supplementary material for: Ensuring sufficient service capacity for removals of long-acting reversible contraceptives: a mixed-method study of provider experiences in Senegal
Source: Gates Open Res. 2022 Apr 8;6:46. [Version 1] doi: 10.12688/gatesopenres.13600.1 (PMC9289255; doi:10.12688/gatesopenres.13600.1)
Supplement: Peer review history

# Peer review: Reviewer response for version 1

**DOI:** 10.21956/gatesopenres.14871.r31967

**Published:** 2022-07-13

**Authors:** Nirali M. Chakraborty

**Affiliations:** 1 Metrics for Management, Oakland, CA, USA

This mixed methods study interviewed public sector Senegalese providers regarding their training and experience in IUD insertion and removal, with a focus on difficult removal situations. The study is well written overall. 

 I offer 2 small comments on the written text, as well as a question for consideration if the data allow (or inclusion as a limitation if they do not). 

1. On page 5, first paragraph, the sentence structure led me to believe that the math presented was incorrect. It is not, but the sentence can be improved by deleting "However, ", in "However, 15% of providers indicated they did not feel confident...".
2. In table 2, do the '-' in the table indicate that there were no or insufficient responses, or that these responses were not offered to the provider for that question?

Related to point 2, I wonder about how this study addressed provider misconceptions related to side effects of IUDs and implants. A study by Chakraborty and colleagues (2015 ^1^) found that trained providers in Nepal had significant misconceptions regarding IUD and implant side effects. If providers were not given the option to list "Weight Gain, Weight loss, Nausea" as side effects of the IUD, then the full picture of provider counseling is not really known. Please address this either in the explanation of table 2, or as a limitation. 

 I look forward to seeing this paper finalized.

Is the work clearly and accurately presented and does it cite the current literature?

Yes

If applicable, is the statistical analysis and its interpretation appropriate?

Not applicable

Are all the source data underlying the results available to ensure full reproducibility?

Yes

Is the study design appropriate and is the work technically sound?

Yes

Are the conclusions drawn adequately supported by the results?

Yes

Are sufficient details of methods and analysis provided to allow replication by others?

Yes

Reviewer Expertise:

Family planning quality, research methodology

I confirm that I have read this submission and believe that I have an appropriate level of expertise to confirm that it is of an acceptable scientific standard.

## References

[1] : Knowledge and perceptions of the intrauterine device among family planning providers in Nepal: a cross-sectional analysis by cadre and sector. *BMC Health Serv Res* .2015;15: 10.1186/s12913-015-0701-y 39 10.1186/s12913-015-0701-y 25627578PMC4322443

# Peer review: Reviewer response for version 1

**DOI:** 10.21956/gatesopenres.14871.r31965

**Published:** 2022-06-23

**Authors:** Rebecca Howett, Chelsea Morroni

**Affiliations:** 1 Botswana Sexual and Reproductive Health Initiative, Gaborone, Botswana

Thank you for the opportunity to review this study. We commend the authors on their comprehensively-conducted and clearly-presented work addressing the important issue of providing LARC services in Senegal.

 We note the perceptive comments made by the first reviewer which we would encourage the authors to address, and our comments are also minor.

1. In the *Methods*, you mention planning to collect data from three districts “representing different geographic and cultural contexts”, but understandably having to cancel work in the third district due to the COVID-19 pandemic. We wonder what effect this had on the representativeness of the study sample relative to Senegal as a whole? For example, are the two included districts both urban whereas the third would have been more rural? To aid those who are unfamiliar with Senegal, we would suggest adding a brief description of the districts to your *Methods *(e.g. urbanicity, significant cultural differences), and mentioning any effect of removing the third district in the limitations paragraph of your *Discussion*.
2. When asking about provider confidence, you sensibly distinguished between 1-rod and 2-rod implants (as shown in *Figure 1*), and we note that there was little difference in the results. In your *Introduction*, please could you clarify how the decision is made between 1-rod versus 2-rod at the time of insertion: do clients get a choice, do providers decide (perhaps opting for the one they are most confident with), or is the decision based on what is available?
3. There is a very minor rounding error in *Table 1*:
  "Timing of last training on implant removal, unsure = 3/55 (5.5%).
  Timing of last training on IUD removal, unsure = 3/55 (5.4%)."
  Please amend the latter to 5.5%.
4. Likewise, there is a very minor rounding error in *Table 5*:
  "Experiences successfully performing difficult removals, IUD embedded in uterine wall = 2/46 (4.4%).
  Experiences referring clients or telling clients to come back due to difficult removals, IUD embedded in uterine wall = 2/46 (4.4%)."
  Please amend both to 4.3%.
5. In the “Management of requests for removal before labeled duration of use” part of the *Qualitative Results *section, you also present data about providers advising removal (i.e. which the client has not requested, and sometimes based on misconceptions about method eligibility criteria). We would suggest splitting this into two separate sub-sections to acknowledge the two different issues.
6. In the next part of the *Qualitative Results *section, consider renaming the “Factors affecting provision of removals” sub-section to “Material factors…”, since the preceding section (or sections if you split it into two, as suggested above) also deals with factors (non-material) that affect the provision of removal.
7. This paper only presents data on provider experiences, however, the *Data availability* section includes links to both provider and client data. Please consider either mentioning in your *Methods* that this work was part of a larger project covering both providers and clients, or removing the references to client data.

Is the work clearly and accurately presented and does it cite the current literature?

Yes

If applicable, is the statistical analysis and its interpretation appropriate?

Yes

Are all the source data underlying the results available to ensure full reproducibility?

Yes

Is the study design appropriate and is the work technically sound?

Yes

Are the conclusions drawn adequately supported by the results?

Yes

Are sufficient details of methods and analysis provided to allow replication by others?

Yes

Reviewer Expertise:

Contraception; implant removal; clinical care

We confirm that we have read this submission and believe that we have an appropriate level of expertise to confirm that it is of an acceptable scientific standard.

# Peer review: Reviewer response for version 1

**DOI:** 10.21956/gatesopenres.14871.r31964

**Published:** 2022-05-16

**Authors:** Megan Christofield

**Affiliations:** 1 Jhpiego, Baltimore, MD, USA

This is an excellent article, capturing a comprehensive picture of LARC removal service availability and provider experiences with removal in Senegal. Congratulations!

 The article is also organized and written exceptionally-well, and thus my comments are few (and minor):

1. First, in your abstract consider adding *voluntarism *or *respecting a user's removal wishes* as an area for improvement. These results come out strongly in your paper.
2. Second, in table 3 I found the row and column labels a bit difficult to interpret. I wonder if you can make it more clear that the data represents the #/% of providers who've had that experience (not the #/% of experiences that have been had by those providers)?
3. Third, in table 5 I wondered whether the 12 providers who've successfully removed an IUD with non-visible strings are a subset of the 15 who've ever had a difficult removal because of non-visible strings. If so, their success rate is actually quite high (12/15) and could be worth representing.
4. Fourth, on page 9 there's a mention of "surgical pliers". I believe this may refer to the forceps used in removal. Consider checking the pliers term for accuracy, as we'd more commonly list these as forceps.
5. Fifth (and final), on page 10 where you say "systemic barriers and potential biases warrant attention as part of a comprehensive approach to support quality removal serves and ensure full, free, and informed choice," consider citing the Senderowicz (2020 ^1^) contraceptive autonomy paper where she makes this point.

Is the work clearly and accurately presented and does it cite the current literature?

Yes

If applicable, is the statistical analysis and its interpretation appropriate?

Yes

Are all the source data underlying the results available to ensure full reproducibility?

Yes

Is the study design appropriate and is the work technically sound?

Yes

Are the conclusions drawn adequately supported by the results?

Yes

Are sufficient details of methods and analysis provided to allow replication by others?

Yes

Reviewer Expertise:

contraceptive access; method choice; LARC removal; self-care

I confirm that I have read this submission and believe that I have an appropriate level of expertise to confirm that it is of an acceptable scientific standard.

## References

[1] : Contraceptive Autonomy: Conceptions and Measurement of a Novel Family Planning Indicator. *Stud Fam Plann* .51(2) : 10.1111/sifp.12114 161-176 10.1111/sifp.12114 32358789
